# Supplementary material for: Demographic changes and behavioural responses shape vulnerability to infectious disease outbreaks
Source: PLoS Comput Biol. 2026 Oct 1;22(10):e1014319. doi: 10.1371/journal.pcbi.1014319 (PMC13652342; doi:10.1371/journal.pcbi.1014319)
Supplement: Supplementary file 1 [file pcbi.1014319.s001.pdf]

**Age group (j)**	**Susceptibility (σ_j) or Infectiousness (τ_j)**			
	Uniform scenario	Linearly increasing scenario	Linearly decreasing scenario	U-shaped scenario
0–4 ($j = 1$)	1.0	0.4	1.6	1.46
5–9 ($j = 2$)	1.0	0.48	1.52	1.17
10–14 ($j = 3$)	1.0	0.56	1.44	0.96
15–19 ($j = 4$)	1.0	0.64	1.36	0.78
20–24 ($j = 5$)	1.0	0.72	1.28	0.78
25–29 ($j = 6$)	1.0	0.8	1.2	0.78
30–34 ($j = 7$)	1.0	0.88	1.12	0.78
35–39 ($j = 8$)	1.0	0.96	1.04	0.78
40–44 ($j = 9$)	1.0	1.04	0.96	0.78
45–49 ($j = 10$)	1.0	1.12	0.88	0.78
50–54 ($j = 11$)	1.0	1.2	0.8	0.78
55–59 ($j = 12$)	1.0	1.28	0.72	0.78
60–64 ($j = 13$)	1.0	1.36	0.64	0.96
65–69 ($j = 14$)	1.0	1.44	0.56	1.17
70–74 ($j = 15$)	1.0	1.52	0.48	1.46
75+ ($j = 16$)	1.0	1.6	0.4	1.76
