# Supplementary material for: Demographic changes and behavioural responses shape vulnerability to infectious disease outbreaks
Source: PLoS Comput Biol. 2026 Oct 1;22(10):e1014319. doi: 10.1371/journal.pcbi.1014319 (PMC13652342; doi:10.1371/journal.pcbi.1014319)
Supplement: Supplementary file 2 [file pcbi.1014319.s004.pdf]

Abbie Evans
Mathematical Institute
University of Oxford
Oxford OX2 6GG

abbie.evans@maths.ox.ac.uk

University of Oxford
Mathematical Institute

28 August 2026

Dear Editors,

Demographic changes and behavioural responses shape vulnerability to infectious disease outbreaks

Thank you for obtaining these reviews and for encouraging us to revise the above-named manuscript. We are grateful to the handling editor and reviewers for their careful reading of the submission and helpful comments.

Please find attached a response in which we itemise and explain the changes that we have made to address the reviewers' comments.

Thank you for handling our manuscript, and we hope that you will now find it appropriate for publication in *PLOS Computational Biology*.

Yours sincerely, on behalf of all authors,

Abbie Evans

Please note: Below, whenever we refer to line numbers in the revised manuscript, these refer to the version of the manuscript with track changes.

Reviewer 1

This paper considers a multi-type branching process model for calculation of outbreak probabilities, used to investigate expected impacts of demographic background and changes from real-world data.

The study appears to be technically correct, however my main concern is a lack of sensitivity analysis to model assumptions. Not every study needs systematic sensitivity investigation, but in this case it is hard to know if the changes found in outbreak probabilities due to demography might be smaller than those due to other factors, meaning that demography is not the most important thing to focus on.

In particular, I think that the model presented is Markov, meaning that offspring distributions will be geometric. However, it should be possible to calculate outbreak probabilities for more general negative binomial offspring distributions as has been done for some time.

Therefore, I would suggest that the authors consider adding some sensitivity analysis to some aspect of model structure in a revised version, but have no other significant concerns about the work.

Response: We thank the reviewer for this helpful suggestion. In our revised manuscript, we have amended the method that we use to calculate the outbreak probability to permit different offspring distributions, enabling us to consider the impact of a negative binomial offspring distribution as suggested (see L106-140). We have also added a sensitivity analysis examining how the population-average probability of a major outbreak varies with the dispersion parameter k (Figure S1B). While the absolute outbreak probability depends on the value of k, the qualitative effect of demographic changes is robust across the range of k values considered, including $k = 1$ which corresponds to the geometric offspring distribution considered in our original submission (L307-311).

Thanks again to the reviewer for this helpful suggestion that has helped us to improve our manuscript.

Reviewer 2

This manuscript reports a mathematical analysis of how demographic change and associated shifts in contact behaviour could influence outbreak probability following importation of a novel pathogen. A range of scenarios are used to examine how outbreak risks are affected by different assumptions about the relationship between age, susceptibility and infectiousness.

The manuscript addresses an important topic, the model design is quite elegant, and the manuscript is (for the most part) clearly written. However, I found that several aspects of the framing, assumptions, and presentation would benefit from clarification, and I make several suggestions below for consideration.

Response: Thank you for your positive assessment of our work, and for your helpful suggestions that have enabled us to improve our manuscript.

While the reported study does have a public health application as its motivation, I also wondered if it might be suited to a mathematically oriented journal, such as Bulletin of Mathematical Biology, Mathematical Biosciences, etc., or even Epidemics.

Response: Thank you for this suggestion. We agree that our manuscript may also be of interest to readers of mathematically oriented journals. However, we believe that *PLOS Computational Biology* is particularly well suited to this work because of its broad interdisciplinary readership. The methodological contributions should be relevant to modellers across a range of computational science disciplines, while the implications of demographic change are of wider interest across the life sciences.

Major comments:

Line 73 and Line 226: South Korea and Nigeria are very different settings in many ways that will affect outbreak risk. Consider rephrasing to describe as a comparison between different demographic scenarios typified by the populations of South Korea and Nigeria, rather than a direct comparison between these two settings, acknowledging that there are cultural, economic and ecological differences that would also have a substantial impact on outbreak risk.

Line 226: As noted above, I feel this comparison would be more appropriately described as a being between different demographic scenarios typified by the demographics of South Korea and Nigeria, rather than a direct comparison between two very different settings.

Response: We agree with the reviewer about this important point. As suggested, we have revised the relevant text to frame our analysis as a comparison between contrasting demographic scenarios typified by the populations of South Korea and Nigeria, rather than as a direct comparison of precise outbreak risk values between the two countries (L78-83, L265-271, L390-393).

Line 200: I'm not sure that I follow the reasoning regarding the scaling of contact rates. If an 10-14 year old in 2020 expects to have (say) 10 daily contacts with other 10-14 year olds, then I don't see why a 10-14 year old in 2050 shouldn't continue to have 10 daily contacts with other 10-14 year olds, even if they represent a smaller fraction of the population (barring catastrophic population collapse). It feels plausible that our intrinsic preference may be for a certain fixed level of social contact, rather than contact with a certain fixed proportion of the population in a particular age group. I'm aware that you explored this alternative as a supplementary analysis; however, I wonder whether it would make sense to reverse the "default" and "supplementary" scenarios?

Response: We agree that maintaining approximately fixed age-specific contact rates as population age structure changes is a plausible alternative assumption. However, because the density correction method has been widely used in previous studies to project contact matrices across settings (see reference [1] at the bottom of this document) and over time (reference [2]), we chose to use this method in the analyses in the main text of our original submission (while also investigating the assumption that the reviewer describes in the Supplementary Information). However, in response to the reviewer's suggestion, we have given the alternative scenario, in which contact networks remain relatively similar over time, greater prominence by incorporating the corresponding results into Figure 2 (see panels G-L) in the main text. We have also emphasised the uncertainty surrounding future contact patterns in the Discussion (L463-466).

Line 270: This feels important, that the observed change in outbreak probability appears to be largely driven by the assumption that people will change their contact preferences in line with age distribution. Age distributions have already shifted markedly in many developed countries over the last century – is there any evidence from these settings that supports this assumption? (There may be – I'm not a demographer – if so, it would be good to cite this.) One context where I can imagine this happening is within households, as the number of contacts among children in that setting shrinks with declining family sizes. However, school class sizes, and other social networks would seem to be more robust (at least among children).

Response: We thank the reviewer for pointing out this important assumption. Empirical evidence about the impact of long-term demographic shifts on contact patterns is unfortunately limited, and longitudinal contact surveys conducted over sufficiently long periods are needed. We have now explicitly acknowledged this assumption in the Discussion, and noted the need for such surveys in future to test this assumption (L467-469). In addition, as noted above, we have included results in which contact networks are assumed to remain similar over time in the main text (Figure 2G-L).

Line 360: While this study focuses on the impact of workforce participation of an ageing population, another factor to consider at least discussing (not necessarily including in the model) is the impact of changing demographics on models of care for aging populations. For example, residential care facilities (or aged care homes) have been associated with increased contact and transmission risk. Declining family sizes (and fewer children to look after aging parents) could result in a greater proportion of the population living in communal settings with increased transmission risk.

Response: We agree that demographic changes may alter models of care for older adults by increasing the proportion of older individuals living in communal settings such as residential care facilities. While the contact structures of residential care facilities differ from those associated with workforce participation, an increase in communal living could similarly increase contact rates among older individuals and therefore increase the outbreak risk. This would be expected to act in the same qualitative direction as the extended workforce participation scenario that we considered, again offsetting the projected reduction in the outbreak probability associated with population ageing. We have added discussion of this possibility and its implications as suggested by the reviewer (L431-435).

Minor comments:

Abstract: While the demographic component of the study was clear, I found the references to behaviour too general in the abstract. I suggest describing this component in more complex terms. For example, contact behaviour, compliance with interventions, health-seeking behaviour? There are so many possibilities! Or is workforce participation the behaviour referred to? If so, I suggest making this more explicit, it only really becomes clear when expanded in the introduction (i.e., line 78).

Response: We thank the reviewer for bringing to our attention that this was unclear. As suggested, we have revised the Abstract to mention extended workforce participation in an ageing society explicitly (L12).

Line 173: Consider using meaningful names for each of scenarios A-D (e.g., as used in lines 276-277).

Response: We have replaced the labels A-D with more descriptive names: uniform, linearly increasing, linearly decreasing and U-shaped (see L202-223).

Line 180 (and elsewhere): In general, methods would be more readable if they consistently referred to the real-world referent (mathematical notation), e.g., “for different age groups (j)”

Response: We have reviewed the Methods and revised the text throughout to ensure that mathematical notation is accompanied by the name of the relevant real-world epidemiological or demographic quantity.

Line 206: Increased workforce participation is certainly a possibility, although by no means a certainty with increasing automation, AI, etc.

Response: We agree that increased workforce participation among older adults is not inevitable, particularly as automation and artificial intelligence may reduce future labour demand or change working arrangements. We have clarified that our analyses reflect a possible future scenario rather than a precise prediction, and noted the potential for increased automation and artificial intelligence to change future contact patterns (L428-430).

Thanks again to the reviewer for their comments that have helped us to improve our manuscript!

References

1. Prem K, Zandvoort K van, Klepac P, Eggo RM, Davies NG, Centre for the Mathematical Modelling of Infectious Diseases COVID-19 Working Group, et al. Projecting contact matrices in 177 geographical regions: An update and comparison with empirical data for the COVID-19 era. PLOS Comput Biol. 2021;17: e1009098. doi:10.1371/journal.pcbi.1009098

2. Weerasuriya CK, Harris RC, McQuaid CF, Gomez GB, White RG. Updating age-specific contact structures to match evolving demography in a dynamic mathematical model of tuberculosis vaccination. PLOS Comput Biol. 2022;18: e1010002. doi:10.1371/journal.pcbi.1010002
