# Supplementary material for: Demographic changes and behavioural responses shape vulnerability to infectious disease outbreaks
Source: PLoS Comput Biol. 2026 Oct 1;22(10):e1014319. doi: 10.1371/journal.pcbi.1014319 (PMC13652342; doi:10.1371/journal.pcbi.1014319)
Supplement: Peer review history

# Peer review: Decision Letter 0

**DOI:** 10.1371/journal.pcbi.1014319.r001

**Authors:** James M McCaw, Denise Kühnert

3 Jul 2026

Demographic changes and behavioural responses shape vulnerability to infectious disease outbreaks

PLOS Computational Biology

Dear Dr. Evans,

Thank you for submitting your manuscript to PLOS Computational Biology. After careful consideration, we feel that it has merit but does not fully meet PLOS Computational Biology's publication criteria as it currently stands. Therefore, we invite you to submit a revised version of the manuscript that addresses the points raised during the review process, particularly the framing and contextual questions raised by reviewer 2 (which are supported by independent comments from reviewer 1 and that imply the need for further modelling and computational work).

Please submit your revised manuscript by Sep 01 2026 11:59PM. If you will need more time than this to complete your revisions, please reply to this message or contact the journal office at ploscompbiol@plos.org. When you're ready to submit your revision, log on to https://www.editorialmanager.com/pcompbiol/ and select the 'Submissions Needing Revision' folder to locate your manuscript file.

Please include the following items when submitting your revised manuscript:

* A letter that responds to each point raised by the editor and reviewer(s). You should upload this letter as a separate file labeled 'Response to Reviewers'. This file does not need to include responses to formatting updates and technical items listed in the 'Journal Requirements' section below.

* A marked-up copy of your manuscript that highlights changes made to the original version. You should upload this as a separate file labeled 'Revised Manuscript with Track Changes'.

* An unmarked version of your revised paper without tracked changes. You should upload this as a separate file labeled 'Manuscript'.

Kind regards,

James M McCaw, PhD

Academic Editor

PLOS Computational Biology

Denise Kühnert

Section Editor

PLOS Computational Biology

**Additional Editor Comments (if provided):**

**Journal Requirements:**

If the reviewer comments include a recommendation to cite specific previously published works, please review and evaluate these publications to determine whether they are relevant and should be cited. There is no requirement to cite these works unless the editor has indicated otherwise.

1) We ask that a manuscript source file is provided at Revision. Please upload your manuscript file as a .doc, .docx, .rtf or .tex. If you are providing a .tex file, please upload it under the item type u2018LaTeX Source Fileu2019 and leave your .pdf version as the item type u2018Manuscriptu2019.

2) Please upload all main figures as separate Figure files in .tif or .eps format. For more information about how to convert and format your figure files please see our guidelines:

https://journals.plos.org/ploscompbiol/s/figures

3) Please upload a copy of Figure Fig 1-5 which you refer to in your text on pages 3, 10, 11, and 14. Or, if the figure is no longer to be included as part of the submission please remove all reference to it within the text.

4) We have noticed that you have uploaded Supporting Information files, but you have not included a list of legends. Please add a full list of legends for your Supporting Information files after the references list.

5) Please amend your detailed Financial Disclosure statement. This is published with the article. It must therefore be completed in full sentences and contain the exact wording you wish to be published.

i) State what role the funders took in the study. If the funders had no role in your study, please state: "The funders had no role in study design, data collection and analysis, decision to publish, or preparation of the manuscript."

ii) If any authors received a salary from any of your funders, please state which authors and which funders..

If you did not receive any funding for this study, please simply state: u201cThe authors received no specific funding for this work.u201d

6) We have amended your Competing Interest statement to comply with journal style. We kindly ask that you double check the statement and let us know if anything is incorrect.

**Reviewers' comments:**

Reviewer's Responses to Questions

**Comments to the Authors:**

**Please note here if the review is uploaded as an attachment.**

Reviewer #1: This paper considers a multi-type branching process model for calculation of outbreak probabilities, used to investigate expected impacts of demographic background and changes from real-world data.

The study appears to be technically correct, however my main concern is a lack of sensitivity analysis to model assumptions. Not every study needs systematic sensitivity investigation, but in this case it is hard to know if the changes found in outbreak probabilities due to demography might be smaller than those due to other factors, meaning that demography is not the most important thing to focus on.

In particular, I think that the model presented is Markov, meaning that offspring distributions will be geometric. However, it should be possible to calculate outbreak probabilities for more general negative binomial offspring distributions as has been done for some time.

Therefore, I would suggest that the authors consider adding some sensitivity analysis to some aspect of model structure in a revised version, but have no other significant concerns about the work.

Reviewer #2: This manuscript reports a mathematical analysis of how demographic change and associated shifts in contact behaviour could influence outbreak probability following importation of a novel pathogen. A range of scenarios are used to examine how outbreak risks are affected by different assumptions about the relationship between age, susceptibility and infectiousness.

The manuscript addresses an important topic, the model design is quite elegant, and the manuscript is (for the most part) clearly written. However, I found that several aspects of the framing, assumptions, and presentation would benefit from clarification, and I make several suggestions below for consideration.

While the reported study does have a public health application as its motivation, I also wondered if it might be suited to a mathematically oriented journal, such as Bulletin of Mathematical Biology, Mathematical Biosciences, etc., or even Epidemics.

Major comments:

Line 73 and Line 226: South Korea and Nigeria are very different settings in many ways that will affect outbreak risk. Consider rephrasing to describe as a comparison between different demographic scenarios typified by the populations of South Korea and Nigeria, rather than a direct comparison between these two settings, acknowledging that there are cultural, economic and ecological differences that would also have a substantial impact on outbreak risk.

Line 200: I’m not sure that I follow the reasoning regarding the scaling of contact rates. If an 10-14 year old in 2020 expects to have (say) 10 daily contacts with other 10-14 year olds, then I don’t see why a 10-14 year old in 2050 shouldn’t continue to have 10 daily contacts with other 10-14 year olds, even if they represent a smaller fraction of the population (barring catastrophic population collapse). It feels plausible that our intrinsic preference may be for a certain fixed level of social contact, rather than contact with a certain fixed proportion of the population in a particular age group. I’m aware that you explored this alternative as a supplementary analysis; however, I wonder whether it would make sense to reverse the “default” and “supplementary” scenarios?

Line 226: As noted above, I feel this comparison would be more appropriately described as a being between different demographic scenarios typified by the demographics of South Korea and Nigeria, rather than a direct comparison between two very different settings.

Line 270: This feels important, that the observed change in outbreak probability appears to be largely driven by the assumption that people will change their contact preferences in line with age distribution. Age distributions have already shifted markedly in many developed countries over the last century – is there any evidence from these settings that supports this assumption? (There may be – I’m not a demographer – if so, it would be good to cite this.) One context where I can imagine this happening is within households, as the number of contacts among children in that setting shrinks with declining family sizes. However, school class sizes, and other social networks would seem to be more robust (at least among children).

Line 360: While this study focuses on the impact of workforce participation of an ageing population, another factor to consider at least discussing (not necessarily including in the model) is the impact of changing demographics on models of care for aging populations. For example, residential care facilities (or aged care homes) have been associated with increased contact and transmission risk. Declining family sizes (and fewer children to look after aging parents) could result in a greater proportion of the population living in communal settings with increased transmission risk.

Minor comments:

Abstract: While the demographic component of the study was clear, I found the references to behaviour too general in the abstract. I suggest describing this component in more complex terms. For example, contact behaviour, compliance with interventions, health-seeking behaviour? There are so many possibilities! Or is workforce participation the behaviour referred to? If so, I suggest making this more explicit, it only really becomes clear when expanded in the introduction (i.e., line 78).

Line 173: Consider using meaningful names for each of scenarios A-D (e.g., as used in lines 276-277)

Line 180 (and elsewhere): In general, methods would be more readable if they consistently referred to the real-world referent (mathematical notation), e.g., “for different age groups (j)”

Line 206: Increased workforce participation is certainly a possibility, although by no means a certainty with increasing automation, AI, etc.

**********

**Have the authors made all data and (if applicable) computational code underlying the findings in their manuscript fully available?**

The PLOS Data policy requires authors to make all data and code underlying the findings described in their manuscript fully available without restriction, with rare exception (please refer to the Data Availability Statement in the manuscript PDF file). The data and code should be provided as part of the manuscript or its supporting information, or deposited to a public repository. For example, in addition to summary statistics, the data points behind means, medians and variance measures should be available. If there are restrictions on publicly sharing data or code —e.g. participant privacy or use of data from a third party—those must be specified.

Reviewer #1: None

Reviewer #2: Yes

**********

PLOS authors have the option to publish the peer review history of their article (what does this mean?). If published, this will include your full peer review and any attached files.

If you choose “no”, your identity will remain anonymous but your review may still be made public.

**Do you want your identity to be public for this peer review?**  For information about this choice, including consent withdrawal, please see our Privacy Policy.

Reviewer #1: No

Reviewer #2: No

[NOTE: If reviewer comments were submitted as an attachment file, they will be attached to this email and accessible via the submission site. Please log into your account, locate the manuscript record, and check for the action link "View Attachments". If this link does not appear, there are no attachment files.]

**Figure resubmission:**

**Reproducibility:**

?>

# Author comment: Author response to Decision Letter 1

**DOI:** 10.1371/journal.pcbi.1014319.r002

31 Aug 2026

Attachment Submitted filename: Response to Reviewers.pdf

# Peer review: Decision Letter 1

**DOI:** 10.1371/journal.pcbi.1014319.r003

**Authors:** James M McCaw, Denise Kühnert

15 Sep 2026

Dear Miss Evans,

We are pleased to inform you that your manuscript 'Demographic changes and behavioural responses shape vulnerability to infectious disease outbreaks' has been provisionally accepted for publication in PLOS Computational Biology.

Before your manuscript can be formally accepted you will need to complete some formatting changes, which you will receive in a follow up email. A member of our team will be in touch with a set of requests.

Please note that your manuscript will not be scheduled for publication until you have made the required changes, so a swift response is appreciated.

IMPORTANT: The editorial review process is now complete. PLOS will only permit corrections to spelling, formatting or significant scientific errors from this point onwards. Requests for major changes, or any which affect the scientific understanding of your work, will cause delays to the publication date of your manuscript.

Should you, your institution's press office or the journal office choose to press release your paper, you will automatically be opted out of early publication. We ask that you notify us now if you or your institution is planning to press release the article. All press must be co-ordinated with PLOS.

Thank you again for supporting Open Access publishing; we are looking forward to publishing your work in PLOS Computational Biology.

Best regards,

James M McCaw, PhD

Academic Editor

PLOS Computational Biology

Denise Kühnert

Section Editor

PLOS Computational Biology

***********************************************************

Reviewer's Responses to Questions

**Comments to the Authors:**

**Please note here if the review is uploaded as an attachment.**

Reviewer #2: Thank you for your consideration and changes to the manuscript. I have only a couple of minor (optional) comments:

(line numbers refer to track changes version)

Line 81: “going forwards” -> “in the future” (“going forwards” sounds like unfortunate corporate speak!)

Fig 3 and S2: a minor point, but the legends in these plots still have scenarios labelled A-D (alongside the descriptive name, which I think reads much more clearly in the text).

**********

**Have the authors made all data and (if applicable) computational code underlying the findings in their manuscript fully available?**

The PLOS Data policy requires authors to make all data and code underlying the findings described in their manuscript fully available without restriction, with rare exception (please refer to the Data Availability Statement in the manuscript PDF file). The data and code should be provided as part of the manuscript or its supporting information, or deposited to a public repository. For example, in addition to summary statistics, the data points behind means, medians and variance measures should be available. If there are restrictions on publicly sharing data or code —e.g. participant privacy or use of data from a third party—those must be specified.

Reviewer #2: Yes

**********

PLOS authors have the option to publish the peer review history of their article (what does this mean?). If published, this will include your full peer review and any attached files.

If you choose “no”, your identity will remain anonymous but your review may still be made public.

**Do you want your identity to be public for this peer review?**  For information about this choice, including consent withdrawal, please see our Privacy Policy.

Reviewer #2: No

# Editor report: Acceptance letter

**DOI:** 10.1371/journal.pcbi.1014319.r004

**Authors:** James M McCaw, Denise Kühnert

PCOMPBIOL-D-26-01122R1

Demographic changes and behavioural responses shape vulnerability to infectious disease outbreaks

Dear Dr Evans,

I am pleased to inform you that your manuscript has been formally accepted for publication in PLOS Computational Biology. Your manuscript is now with our production department and you will be notified of the publication date in due course.

The corresponding author will soon be receiving a typeset proof for review, to ensure errors have not been introduced during production. Please review the PDF proof of your manuscript carefully, as this is the last chance to correct any errors. Please note that major changes, or those which affect the scientific understanding of the work, will likely cause delays to the publication date of your manuscript.

Soon after your final files are uploaded, unless you have opted out, the early version of your manuscript will be published online. The date of the early version will be your article's publication date. The final article will be published to the same URL, and all versions of the paper will be accessible to readers.

For Research, Software, and Methods articles, you will receive an invoice from PLOS for your publication fee after your manuscript has reached the completed accept phase. If you receive an email requesting payment before acceptance or for any other service, this may be a phishing scheme. Learn how to identify phishing emails and protect your accounts at https://explore.plos.org/phishing.

Thank you again for supporting PLOS Computational Biology and open-access publishing. We are looking forward to publishing your work!

With kind regards,

Sharmila Kamatchi

PLOS Computational Biology | Carlyle House, Carlyle Road, Cambridge CB4 3DN | United Kingdom ploscompbiol@plos.org | Phone +44 (0) 1223-442824 | ploscompbiol.org | @PLOSCompBiol
